# Early Postoperative Predictors of 30-Day Mortality After Pediatric Liver Transplantation: A Trajectory-Based Analysis

**DOI:** 10.3390/jcm15114385

**Published:** 2026-06-05

**Authors:** Ibrahim Bingol, Tonguc Utku Yilmaz, Ozge Umur, Guntulu Sık, Hamdi Karakayalı, Agop Citak

**Affiliations:** 1 Department of Pediatric Intensive Care, Acıbadem Mehmet Ali Aydınlar University, 34638 Istanbul, Turkey; ibrahimbingol@gmail.com (I.B.); ozgeumur00@gmail.com (O.U.); drguntulu@hotmail.com (G.S.); agopcitak@hotmail.com (A.C.); 2 Liver Transplantation Unit, Acıbadem Mehmet Ali Aydınlar University, 34638 Istanbul, Turkey; karakayali.hamdi@gmail.com

**Keywords:** pediatric liver transplantation, 30-day mortality, PRISM-III, INR×lactate, lactate-to-albumin ratio, biomarker trajectory, internal validation

## Abstract

**Background/Objectives**: Early mortality after pediatric liver transplantation remains a clinical challenge, yet few studies have specifically addressed 30-day outcomes. Conventional pretransplant scores such as the age-appropriate MELD/PELD score were not designed for post-transplant risk prediction. We aimed to evaluate whether dynamic postoperative biomarker trajectories and novel composite ratios can identify high-risk patients. **Methods**: This single-center retrospective cohort study included 140 consecutive pediatric patients (<18 years) who underwent primary liver transplantation between 2015 and 2023. Patients were classified as deceased (≤30 days, *n* = 11) or survivors (>30 days, *n* = 129). PRISM-III, PELOD-2, and age-appropriate MELD/PELD scores were evaluated. Serial laboratory parameters were collected at pretransplant and at 0, 24, and 72 h. Delta (Δ) values and composite ratios—including lactate clearance, lactate-to-albumin ratio (LAR), INR×lactate product, platelet ratio, and fibrinogen/INR—were calculated. Penalized logistic regression (Firth method) was used for multivariate analysis. Internal validation was performed using bootstrap resampling (1000 iterations) and leave-one-out cross-validation (LOO-CV). Because two of the three components of the multivariable model (ΔINR, ΔALT) were derived from 72-h values, the model is best understood as a 72-h landmark risk model rather than as an immediate post-transplant early-warning tool. **Results**: The 30-day mortality rate was 7.9% (11/140), with central nervous system complications as the leading cause (36.4%). PRISM-III demonstrated excellent discrimination (AUROC 0.957; cut-off ≥ 14); the age-appropriate MELD/PELD score, a pretransplant tool not designed for post-transplant prediction, showed near-chance performance (AUROC 0.513; *p* = 0.576). A distinctive biomarker crossover pattern was observed: non-survivors had paradoxically lower pretransplant INR, ALT, and LAR values, but trajectories diverged sharply by 24 h. The INR×lactate product achieved an AUROC of 0.981 at 72 h. LAR at 24 h achieved 0.909, and lactate clearance at 0 → 72 h achieved 0.783. Postoperative hypernatremia emerged as a strong predictor (AUROC 0.884). In multivariate analysis, PRISM-III (OR 4.00), ΔINR (OR 3.28), and ΔALT (OR 3.46) were independent predictors (apparent AUROC 0.989). Internal validation confirmed model stability: bootstrap-corrected AUROC was 0.978; LOO-CV AUROC was 0.957 (sensitivity 90.9%, specificity 96.9%). **Conclusions**: Dynamic postoperative factors—rather than pretransplant disease severity—appeared more strongly associated with 30-day mortality after pediatric liver transplantation in this single-center exploratory analysis. The INR×lactate product, a novel two-variable composite, showed very high apparent discrimination (AUROC 0.981) and is proposed as a hypothesis-generating candidate marker requiring prospective external validation before any clinical use. The combined PRISM-III + ΔINR + ΔALT model (best understood as a 72-h landmark risk model, since two of its three components are defined at 72 h postoperatively) demonstrated robust internal validation performance (LOO-CV AUROC 0.957); however, given the small number of events (*n* = 11) and the absence of external validation, the model should be regarded as exploratory.

## 1. Introduction

Liver transplantation remains the only curative treatment for children with end-stage liver disease, acute liver failure, and selected metabolic disorders. Contemporary registry data demonstrate one-year patient survival exceeding 90% in experienced centers [1,2]. These gains are largely attributable to reductions in early postoperative mortality, yet the first 30 days remain the highest-risk interval, with a concentration of graft-related catastrophes, multi-organ dysfunction, and fulminant infection [3,4].

Current risk stratification relies on the age-appropriate Model for End-Stage Liver Disease/Pediatric End-Stage Liver Disease (MELD/PELD) score, which was designed for waitlist mortality prediction and underestimates pretransplant risk by up to 17% [5,6]. Its predictive value for post-transplant 30-day mortality has not been systematically evaluated. The PRISM-III score captures dynamic physiological derangement within the first 24 h of intensive care unit (ICU) admission (pooled AUROC 0.84) [7,8], but its role in pediatric liver transplantation remains controversial, with studies reporting both significant [9] and non-significant [10] associations with outcomes.

Beyond static scores, serial biomarker monitoring captures the trajectory of graft recovery or deterioration. Dynamic prediction models incorporating INR, bilirubin, and platelet trends have improved AUROC from 0.784 to 0.887 in pediatric acute liver failure [11]. Lactate clearance is of particular interest in the transplant setting, as hepatic lactate metabolism directly reflects graft function; in adult liver transplantation, early lactate clearance predicts initial poor graft function with an AUROC of 0.961 [12]. More recently, composite ratios such as the lactate-to-albumin ratio (LAR) have emerged as powerful prognostic tools in pediatric critical care, outperforming individual biomarkers for mortality and organ dysfunction prediction [13,14]. However, neither serial trajectory analysis, lactate clearance, nor composite ratios has been evaluated for early mortality prediction after pediatric liver transplantation.

Few studies have specifically addressed 30-day mortality in this population. Cho et al. identified preoperative ICU admission and reoperation as independent predictors in 101 infants [3], and Ciria et al. developed a composite score for 30-day graft loss in 422 recipients (AUROC 0.774) [15]. To our knowledge, this is the first single-center pediatric liver transplantation cohort in which (i) the postoperative PRISM-III score is statistically assessed alongside the pretransplant age-appropriate MELD/PELD score (DeLong test) for 30-day post-transplant mortality, (ii) trajectory-based delta values (ΔINR, ΔALT) are evaluated as independent predictors of early mortality, (iii) the INR×lactate product is proposed as a candidate composite marker, and (iv) the lactate-to-albumin ratio (LAR) is examined trajectorially.

We sought to: (1) assess whether the pretransplant age-appropriate MELD/PELD score retains predictive performance for 30-day post-transplant mortality, and how it compares with the postoperative PRISM-III score; (2) characterize serial biomarker trajectories and determine whether delta values and composite ratios—including lactate clearance, LAR, and INR×lactate—independently predict early mortality; and (3) develop and internally validate a combined predictive model using bootstrap resampling and leave-one-out cross-validation.

## 2. Materials and Methods

### 2.1. Study Design and Setting

This single-center retrospective cohort study was conducted at Acıbadem Mehmet Ali Aydınlar University Hospital in Istanbul, Turkey—a high-volume living-donor liver transplantation center. All consecutive pediatric patients (<18 years) who underwent primary liver transplantation between January 2015 and December 2023 were screened. The study was approved by the Acıbadem University Ethics Committee (No: 2026-02/51), with a waiver of informed consent granted given the retrospective design. Reporting follows the Strengthening the Reporting of Observational Studies in Epidemiology (STROBE) guidelines [16].

### 2.2. Patient Selection

Inclusion criteria were: (i) age < 18 years at transplantation, (ii) primary (first) liver transplantation, and (iii) availability of the primary outcome data. Patients who underwent multi-organ transplantation, retransplantation, or had incomplete 30-day follow-up were excluded.

### 2.3. Outcome Definitions

The primary outcome was 30-day all-cause mortality, defined as death from any cause within 30 days of transplantation (day of surgery = day 0), consistent with the definition used by Cho et al. [3]. Patients were classified as deceased (≤30 days) or survivors (>30 days). The survivor group included both living patients and those who died after 30 days, as these patients had survived the primary endpoint interval. This landmark-style classification was prespecified to preserve a strict 30-day binary endpoint, which is methodologically aligned with the prognostic question (early vs. late mortality) and avoids confounding the model with deaths driven by long-term complications such as chronic rejection or post-transplant lymphoproliferative disease. To address potential bias from this classification choice, an exploratory comparison of early versus late deaths is presented in Section 3.11 and Supplementary Table S1.

### 2.4. Data Collection

Recipient variables included age at transplantation, sex, body weight, primary liver disease, and pretransplant disease severity assessed by the age-appropriate MELD/PELD score, with the Pediatric End-Stage Liver Disease (PELD) score calculated for patients younger than 12 years [5]; the Model for End-Stage Liver Disease (MELD) score was used for patients aged ≥ 12 years. Donor age and sex were recorded. Operative variables included operation duration. Preoperative clinical status was documented including mechanical ventilation, vasopressor use, and ICU admission prior to transplantation.

For simplicity of presentation, and consistent with the observation that all 30-day deaths occurred in the PELD-scored subgroup (*n* = 128), we refer to this variable as “PELD” throughout the manuscript. A sensitivity analysis restricted to PELD-scored patients yielded near-identical results (AUROC 0.524 in PELD-only analysis vs. 0.513 in primary analysis).

Postoperative variables included mechanical ventilation duration, pediatric intensive care unit (PICU) and hospital length of stay, requirement for continuous renal replacement therapy (CRRT), extracorporeal membrane oxygenation (ECMO), and therapeutic plasma exchange (TPE). Complications recorded were primary nonfunction (per United Network for Organ Sharing (UNOS) criteria), acute rejection (diagnosed by clinical suspicion, with biopsy confirmation when feasible), hepatic artery thrombosis, portal vein thrombosis, biliary leak, and sepsis. Cause and day of death were documented for all deceased patients.

Missing data were infrequent (<5% for any key variable) and were handled using complete-case analysis; no imputation was performed. Sepsis was operationally defined as clinical deterioration (new-onset or worsening of hemodynamic, respiratory, or organ dysfunction) prompting antimicrobial escalation, with or without a positive microbiological culture, adapted from the 2005 International Pediatric Sepsis Consensus Conference (IPSCC) criteria [17].

### 2.5. Postoperative Severity Scores

The PRISM-III (Pediatric Risk of Mortality III) score was calculated from the worst physiological parameters recorded within the first 24 h of PICU admission, as originally described by Pollack et al. [7]. The PELOD-2 (Pediatric Logistic Organ Dysfunction 2) score was calculated from the same time window [18].

### 2.6. Standardized Center Practices

*Donor source and surgery.* All 140 transplants were performed using ABO-compatible living-related donor grafts by a single dedicated pediatric liver transplantation surgical team using a standardized institutional technique throughout the study period.

*Immunosuppression.* All recipients received a uniform protocol that remained unchanged throughout the study period: basiliximab induction on day 0 and day 4 (10 mg for patients ≤ 35 kg; 20 mg for patients > 35 kg) with an intraoperative methylprednisolone bolus, followed by maintenance with tacrolimus (target trough approximately 8–10 ng/mL during the early postoperative months and tapered thereafter), mycophenolate mofetil (15–20 mg/kg/dose twice daily), and prednisolone (2 mg/kg/day, tapered over 3 to 6 months). Standardized infectious prophylaxis with trimethoprim-sulfamethoxazole and fluconazole was given to all recipients.

*Postoperative supportive care.* Decisions to initiate continuous renal replacement therapy (CRRT) and therapeutic plasma exchange (TPE) were made by the treating pediatric intensivist on a case-by-case basis, guided by clinical indications rather than fixed numerical thresholds. CRRT was typically initiated for refractory fluid overload, severe metabolic derangement, or uncontrolled hyperammonemia; TPE was typically initiated for antibody-mediated rejection, severe coagulopathy, or selected cases of early allograft dysfunction. Hypertonic saline (3%) was used for clinically suspected cerebral edema. The case-by-case nature of these decisions is acknowledged as a source of inter-clinician variability in Section 4.11.

### 2.7. Serial Laboratory Measurements

A comprehensive panel of laboratory parameters was collected at four standardized time points: pretransplant (within 24 h before surgery) and 0, 24, and 72 h postoperatively. Parameters included international normalized ratio (INR), albumin, aspartate aminotransferase (AST), alanine aminotransferase (ALT), total bilirubin, lactate, creatinine, platelet count, fibrinogen, C-reactive protein (CRP), procalcitonin, sodium, and complete blood count with differential (neutrophil, lymphocyte counts). The 0-h time point was operationally defined as the first laboratory sample drawn upon PICU admission immediately following intraoperative hand-off from the anesthesia team (typically within 30 min of PICU arrival). The 24-h and 72-h samples were drawn during routine morning rounds and were anchored to the PICU admission time rather than the calendar day, ensuring consistent inter-sample intervals across patients. Perioperative blood product transfusions (red blood cells, fresh frozen plasma, platelets), intraoperative crystalloid and colloid resuscitation, and hypertonic saline administration were not included as adjustment covariates in the multivariate model.

### 2.8. Delta Values

To quantify the direction and magnitude of postoperative change, delta (Δ) values were calculated as the difference between the 72-h and pretransplant measurements. For transaminases, percentage change was used: ΔALT(%) = [(ALT72h − ALTpreop)/ALTpreop × 100]. For INR, absolute change was calculated: ΔINR = INR72h − INRpreop. Positive delta values indicate worsening (rising transaminases or INR); negative values indicate improvement.

### 2.9. Composite Biomarker Ratios

Several composite ratios were calculated at each available time point to integrate complementary pathophysiological domains into single metrics.

Lactate clearance was defined as [(lactate0h − lactate24h)/lactate0h × 100], expressed as a percentage, with positive values indicating effective hepatic clearance. This metric reflects graft metabolic function, as the liver is the principal site of lactate metabolism [12].

The lactate-to-albumin ratio (LAR) was calculated as lactate (mmol/L) divided by albumin (g/dL) at each time point. LAR integrates tissue perfusion adequacy (lactate) with inflammatory burden and hepatic synthetic reserve (albumin) [13,14].

The INR×lactate product was calculated at 24 and 72 h as a composite index of coagulation dysfunction and perfusion impairment, combining graft synthetic function (INR) with metabolic clearance capacity (lactate).

The platelet ratio was defined as platelet count at 72 h divided by the pretransplant value, reflecting platelet consumption, sequestration, and thrombopoietic recovery. The fibrinogen-to-INR ratio was calculated at 24 and 72 h as an index of coagulation recovery relative to synthetic dysfunction.

### 2.10. Statistical Analysis

Continuous variables were assessed for normality using the Shapiro–Wilk test and are presented as median (interquartile range). Categorical variables are expressed as n (%). Comparisons between the deceased and survivor groups used the Mann–Whitney U test for continuous variables and Fisher’s exact test for categorical variables. A two-sided *p*-value < 0.05 was considered statistically significant.

Receiver operating characteristic (ROC) curves were constructed for each predictor, and the area under the curve (AUROC) was calculated. Optimal cut-off values were determined by maximizing the Youden index (sensitivity + specificity − 1). Pairwise comparison of AUROCs was performed using the DeLong test [19]. The 95% confidence intervals for individual AUROC values were computed using the DeLong method, and 95% confidence intervals for sensitivity and specificity were derived using the Wilson score method. We explicitly note that all reported cut-off values for individual biomarkers and composite ratios (including INR×lactate, lactate-to-albumin ratio [LAR], and lactate clearance) were derived *post hoc* from the present cohort using the Youden index and were not prespecified from prior literature. These cut-offs are therefore data-driven and require external validation before clinical use. Given that multiple biomarkers were evaluated in parallel, a Bonferroni-adjusted significance threshold (*p* < 0.05/k, where k is the number of comparisons) was considered as a sensitivity check; however, all primary findings reported as significant maintained *p* < 0.001 and remained robust to such adjustment. Nevertheless, the absence of a single pre-specified primary biomarker is acknowledged as a source of multiple-testing bias (see Section 4.11).

For multivariate analysis, Firth’s penalized logistic regression was employed to address the small number of outcome events [20]. This method reduces the bias inherent in maximum likelihood estimation when the events-per-variable (EPV) ratio is low [21]. Given 11 events, a maximum of 3 predictor variables was permitted in the final model (EPV ≥ 3.7). Variables were selected based on clinical relevance, univariate significance (*p* < 0.05), and the absence of collinearity (variance inflation factor < 5). Odds ratios with 95% confidence intervals are reported.

### 2.11. Internal Validation

To quantify potential overfitting and assess model stability, two complementary internal validation strategies were performed [22]. First, bootstrap resampling with 1000 iterations was conducted. In each iteration, a bootstrap sample was drawn with replacement from the original dataset, the model was fitted on the bootstrap sample, and the AUROC was calculated on both the bootstrap sample and the original dataset. The mean optimism (difference between bootstrap and original performance) was subtracted from the apparent AUROC to yield the optimism-corrected AUROC.

Second, leave-one-out cross-validation (LOO-CV) was performed. Each patient was sequentially excluded, the model was trained on the remaining 139 patients, and the excluded patient’s probability of mortality was predicted. LOO-CV AUROC, sensitivity, and specificity were calculated from these cross-validated predictions, providing an unbiased estimate of true predictive performance. Formal calibration metrics (Hosmer–Lemeshow goodness-of-fit, Brier score, smoothed calibration plot) were not computed, as the small number of outcome events (*n* = 11) does not allow reliable partitioning of the predicted-probability space [22]. Internal validation was therefore restricted to assessment of discrimination.

### 2.12. Survival Analysis

Thirty-day survival was estimated using the Kaplan–Meier method, with subgroup comparisons performed by the log-rank test. Stratification variables included PRISM-III (≥14 vs. <14), ΔINR (≥0.4 vs. <0.4), and concurrent CRRT plus TPE requirement.

For predictors derived from 72-h postoperative measurements (ΔINR, Δ72 h values), survival analyses were interpreted within a 72-h landmark framework: patients who died within the first 72 h (*n* = 0) could not be stratified by 72-h variables, and analyses reflect conditional survival from the landmark time onward. The CRRT + TPE combination was analyzed descriptively rather than in Kaplan–Meier analysis because these interventions are time-varying and are initiated in response to deterioration rather than at baseline.

All analyses were performed using SPSS version 26.0 (IBM Corporation, Armonk, NY, USA) and R version 4.3.0 (R Foundation for Statistical Computing, Vienna, Austria). R packages included logistf for penalized regression, pROC for ROC analysis, boot for bootstrap validation, and survival for Kaplan–Meier analysis.

## 3. Results

### 3.1. Patient Characteristics

**Figure 1:**
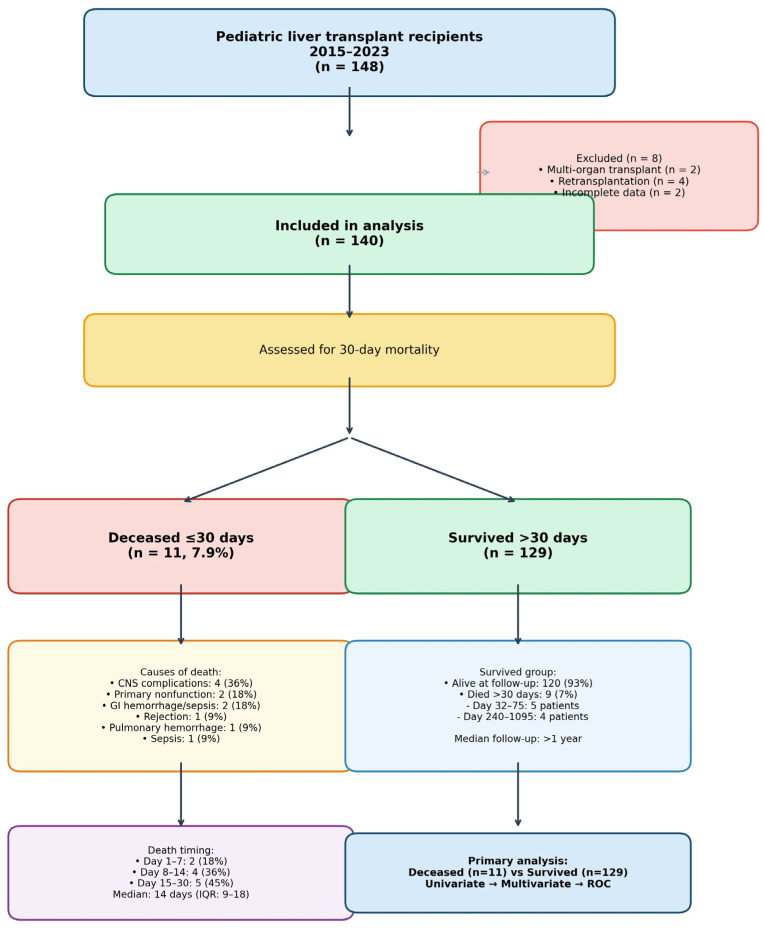
Patient flow diagram. Of 140 consecutive pediatric liver transplant recipients screened during the study period (2015–2023), all met inclusion criteria. Patients were classified as deceased within 30 days (*n* = 11, 7.9%) or survived beyond 30 days (*n* = 129, 92.1%). The survivor group included 120 living patients and 9 patients who died after 30 days.

During the study period, 140 pediatric patients met the inclusion criteria (Figure 1). The median age was 22 months (IQR: 11–70), with 73 females (52.1%) and 67 males (47.9%). The median weight was 10.0 kg (IQR: 7.8–20.0). Biliary atresia was the most common indication (45.7%), followed by metabolic diseases (22.1%) and progressive familial intrahepatic cholestasis (11.4%).

### 3.2. Thirty-Day Mortality

Thirty-day mortality occurred in 11 patients (7.9%). The median time to death was 14 days (IQR: 8–17). Two patients (18.2%) died within 7 days, four (36.4%) between days 8–14, and five (45.5%) between days 15–30. Central nervous system complications were the leading cause of death (4/11, 36.4%), including brain death in three patients and intracranial hemorrhage with sepsis in one. Other causes included primary nonfunction (2/11, 18.2%), gastrointestinal hemorrhage with sepsis (2/11, 18.2%), acute rejection (1/11, 9.1%), pulmonary hemorrhage (1/11, 9.1%), and sepsis (1/11, 9.1%). Overall mortality (all time points) was 14.3% (20/140).

### 3.3. Univariate Analysis of Conventional Variables

Baseline recipient and transplant-associated characteristics are presented in Table 1. No significant differences were observed in age (*p* = 0.464), weight (*p* = 0.214), or age-appropriate MELD/PELD score (*p* = 0.576) between groups. Preoperative INR was paradoxically lower in the deceased group (1.2 vs. 1.7; *p* = 0.003), as was the neutrophil-to-lymphocyte ratio (1.2 vs. 2.6; *p* = 0.001). Operation duration was shorter in non-survivors (465 vs. 532 min; *p* = 0.016).

Postoperative variables (Table 2) revealed markedly higher PRISM-III scores in non-survivors (15.0 vs. 8.0; *p* < 0.001). PELOD-2 scores were also elevated (7.0 vs. 4.0; *p* = 0.046). Requirements for organ support were substantially greater in the deceased group, including CRRT (81.8% vs. 17%; *p* < 0.001), TPE (90.9% vs. 26.4%; *p* < 0.001), and prolonged mechanical ventilation (14 vs. 4 days; *p* < 0.001). Acute rejection was significantly more frequent (45.5% vs. 7.8%; *p* = 0.002). Among 15 patients with acute rejection, 30-day mortality was 33.3% compared to 4.8% without rejection (*p* = 0.002).

### 3.4. Serial Biomarker Trajectories

**Figure 2:**
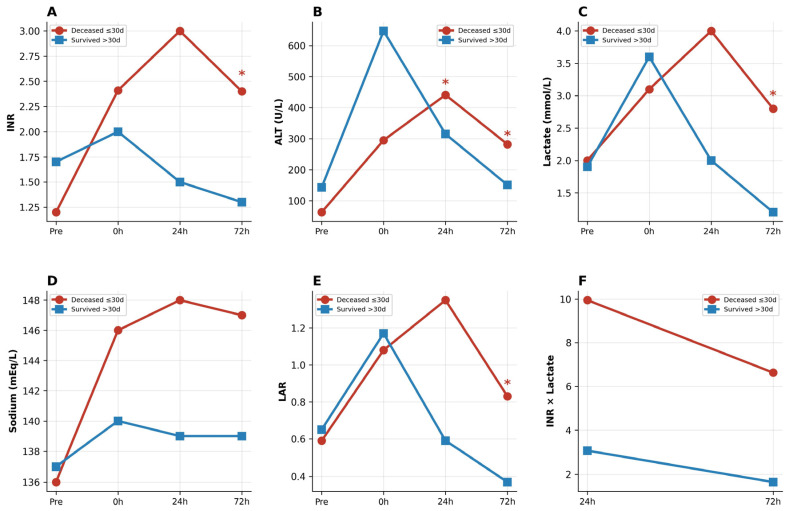
Serial biomarker trajectories in deceased (≤30 days, *n* = 11; red circles) versus survivor (>30 days, *n* = 129; blue squares) groups. Values represent medians at each time point (pretransplant, 0, 24, and 72 h postoperatively). Asterisks indicate statistically significant differences (*p* < 0.05, Mann–Whitney U test). (**A**) INR trajectory demonstrating crossover at 24 h. (**B**) ALT trajectory with analogous crossover pattern. (**C**) Lactate trajectory diverging at 24 h. (**D**) Sodium trajectory showing persistent hypernatremia in non-survivors from 0 h onward. (**E**) Lactate-to-albumin ratio (LAR) trajectory with crossover at 24 h. (**F**) INR×lactate product at 24 and 72 h.

A distinctive crossover pattern was observed across multiple biomarkers (Figure 2). At the pretransplant time point, INR and ALT values were paradoxically lower in non-survivors than survivors. By 24 h, trajectories diverged sharply: non-survivors demonstrated progressive worsening while survivors recovered. INR at 24 h was 3.0 in the deceased group versus 1.5 in survivors (*p* < 0.001); by 72 h, the difference persisted (2.4 vs. 1.3; *p* < 0.001). Lactate diverged significantly at 24 h (4.0 vs. 2.0 mmol/L; *p* < 0.001). Delta values (Table 3) demonstrated that ΔALT was +320.9% in deceased versus +9.8% in survived (*p* < 0.001), and ΔINR was +1.1 versus −0.4 (*p* < 0.001).

### 3.5. Postoperative Hypernatremia

A striking finding was the development of persistent hypernatremia in non-survivors (Figure 2D). Pretransplant sodium levels were comparable between groups (136 vs. 137 mEq/L; *p* = 0.883). However, by 24 h, non-survivors demonstrated significantly elevated sodium (148 vs. 139 mEq/L; *p* < 0.001), which persisted at 72 h (147 vs. 139 mEq/L; *p* < 0.001; AUROC 0.884).

### 3.6. Composite Biomarker Ratios

The INR×lactate product emerged as the strongest exploratory composite predictor in this cohort (Table 3). At 24 h, INR×lactate was 9.99 in deceased versus 3.1 in survivors (*p* < 0.001; AUROC 0.965), and at 72 h, it was 6.63 versus 1.65 (*p* < 0.001; AUROC 0.981). This composite integrates graft synthetic function (INR) with metabolic clearance capacity (lactate) into a single data-driven composite metric. The cut-off thresholds reported here (INR×lactate > 4.8 at 24 h, >3.3 at 72 h) were not pre-specified from prior literature but were derived *post hoc* from the present cohort using the Youden index, and they are therefore inherently optimistic for the same dataset. They should not be interpreted as ready-to-use clinical thresholds. These composite biomarkers were evaluated as exploratory indices.

The lactate-to-albumin ratio (LAR) demonstrated a trajectory crossover pattern analogous to that observed for INR and ALT. Pretransplant LAR was similar between groups (0.59 vs. 0.65; *p* = 0.690). By 24 h, LAR was markedly elevated in non-survivors (1.35 vs. 0.59; *p* < 0.001; AUROC 0.909), and this difference persisted at 72 h (0.83 vs. 0.37; *p* < 0.001; AUROC 0.907). These trajectory-based ratios should be regarded as hypothesis-generating exploratory findings pending external validation.

Lactate clearance from 0 to 24 h was negative in non-survivors (−25.0%), indicating worsening lactate accumulation, compared to +47.1% clearance in survivors (*p* = 0.014). By 72 h, non-survivors achieved only 12.9% clearance versus 65.9% in survivors (*p* = 0.002; AUROC 0.783). The platelet ratio (72 h/pretransplant) was significantly lower in non-survivors (0.43 vs. 0.78; *p* = 0.002; AUROC 0.786), reflecting impaired thrombopoietic recovery. The fibrinogen-to-INR ratio was markedly lower in deceased patients at both 24 h (41.6 vs. 132.2; *p* < 0.001; AUROC 0.891) and 72 h (91.9 vs. 203.7; *p* < 0.001; AUROC 0.888).

### 3.7. Combined Extracorporeal Support

Among 17 patients who received both CRRT and TPE, 30-day mortality was 52.9% (9/17) compared to 1.2% (1/82) in those receiving neither modality (*p* < 0.001). This concurrent requirement for dual extracorporeal support was associated with high short-term mortality in descriptive analysis.

### 3.8. Multivariate Analysis and Internal Validation

Based on clinical relevance, univariate significance, and absence of collinearity, three variables were entered into the penalized logistic regression model (Table 4): PRISM-III, ΔINR, and ΔALT(%). All three remained independently significant: PRISM-III (OR 4.00; 95% CI 1.82–8.79; *p* = 0.001), ΔINR (OR 3.28; 95% CI 1.56–6.89; *p* = 0.002), and ΔALT (OR 3.46; 95% CI 1.64–7.30; *p* = 0.001). The apparent AUROC of the combined model was 0.989 (95% CI 0.971–1.000), with sensitivity of 100% and specificity of 93.8%.

Internal validation confirmed model stability. Bootstrap resampling (1000 iterations) yielded a mean optimism of 0.011, resulting in an optimism-corrected AUROC of 0.978. Leave-one-out cross-validation demonstrated an AUROC of 0.957, with sensitivity of 90.9% and specificity of 96.9%. The minimal difference between the apparent (0.989) and LOO-CV (0.957) AUROCs indicates that optimism in the model was limited, although external validation remains essential.

### 3.9. ROC Analysis

**Figure 3:**
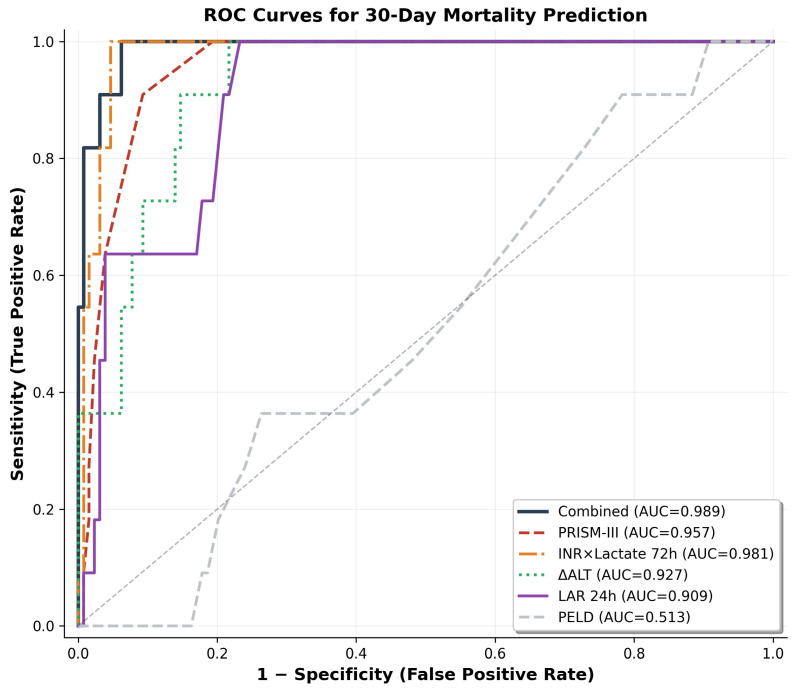
Receiver operating characteristic (ROC) curves for 30-day mortality prediction. The combined model (PRISM-III + ΔINR + ΔALT; apparent AUROC 0.989; black solid line) is compared with: INR×lactate at 72 h (AUROC 0.981; orange dashed), PRISM-III (AUROC 0.957; red dashed), ΔALT (AUROC 0.927; green dotted), LAR at 24 h (AUROC 0.909; purple solid), and age-appropriate MELD/PELD (AUROC 0.513; gray dashed). DeLong test: PRISM-III vs. age-appropriate MELD/PELD, *p* < 0.001. The diagonal grey dashed line represents the line of no discrimination (AUROC = 0.5, corresponding to random chance).

Individual predictor performance is summarized in Table 5 and Figure 3. Among conventional scores, PRISM-III demonstrated excellent discrimination (AUROC 0.957; optimal cut-off ≥ 14: sensitivity 90.9%, specificity 90.7%). PELOD-2 showed moderate performance (AUROC 0.681). The age-appropriate MELD/PELD score, designed for pretransplant listing rather than post-transplant prediction, showed near-chance performance (AUROC 0.513; *p* = 0.576). DeLong comparison showed that the postoperative PRISM-III score discriminated 30-day mortality significantly better than the pretransplant age-appropriate MELD/PELD score (*p* < 0.001) and better than PELOD-2 (*p* = 0.002); these comparisons should be interpreted as evidence of the limitations of pretransplant tools for postoperative outcome prediction rather than as a contest between equivalent instruments.

Among composite ratios, INR×lactate at 72 h achieved the highest individual AUROC (0.981), followed by INR×lactate at 24 h (0.965), ΔALT (0.927), LAR at 24 h (0.909), ΔINR (0.908), sodium at 72 h (0.884), and fibrinogen/INR at 24 h (0.891). Notably, the simple INR×lactate product at 72 h approached the apparent performance of the three-variable combined model (0.981 vs. 0.989), warranting prospective external validation as an exploratory candidate marker before any clinical use.

### 3.10. Survival Analysis

**Figure 4:**
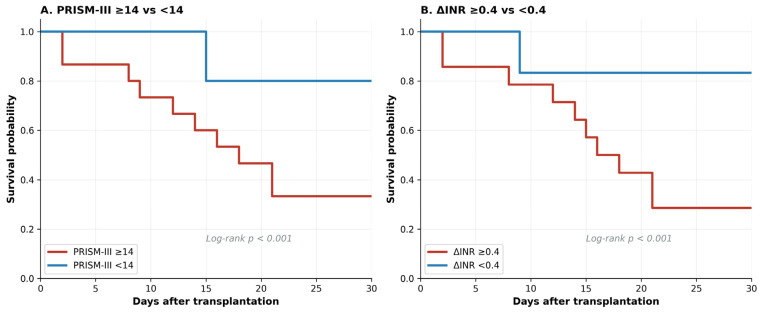
Kaplan–Meier survival curves for 30-day mortality. (**A**) Stratification by PRISM-III score: ≥14 (red, *n* = 14; 30-day survival 57.1%) versus <14 (blue, *n* = 126; 30-day survival 98.3%). Log-rank *p* < 0.001; (**B**) 72-h landmark analysis stratified by ΔINR: ≥0.4 (red; 30-day survival 41.7%) versus <0.4 (blue; 30-day survival 98.4%). Log-rank *p* < 0.001. Panel (**B**) is a landmark analysis, as ΔINR is defined at the 72-h time point.

Kaplan–Meier analysis stratified by PRISM-III ≥ 14 versus <14 demonstrated 30-day survival of 57.1% versus 98.3% (log-rank *p* < 0.001; Figure 4A). In a 72-h landmark analysis, stratification by ΔINR ≥ 0.4 versus <0.4 showed survival of 41.7% versus 98.4% (log-rank *p* < 0.001; Figure 4B).

### 3.11. Exploratory Analysis: Early Versus Late Mortality Patterns

An exploratory comparison of early (≤30 days, *n* = 11) versus late (>30 days, *n* = 9) deaths revealed two distinct pathophysiological patterns (Supplementary Table S1). Early deaths were characterized by acute catastrophic events: CNS complications (36.4%) and primary nonfunction (18.2%) predominated, with acute rejection in 45% of cases. Late deaths were driven by prolonged sepsis (78%), with significantly longer mechanical ventilation (27 vs. 14 days; *p* = 0.017) and hospital stays (45 vs. 23 days; *p* = 0.016). Coagulation at 72 h was significantly worse in early deaths (INR 2.4 vs. 1.6; *p* = 0.001), and sodium was higher (147 vs. 142 mEq/L; *p* = 0.039), consistent with more severe acute multi-organ dysfunction. These exploratory findings suggest that the predictive model—which captures early coagulation failure and hepatic dysfunction—appears to align with the acute catastrophic pattern of early mortality, although this observation is hypothesis-generating and requires confirmation in independent cohorts.

## 4. Discussion

In this exploratory single-center cohort of 140 pediatric liver transplant recipients, dynamic postoperative factors appeared to be more informative for 30-day mortality risk than pretransplant severity scores, and several composite biomarkers showed high apparent discrimination. Five key findings emerged: (1) PRISM-III, a postoperative physiologic severity score, showed strong discrimination (apparent AUROC 0.957) for 30-day mortality, whereas the pretransplant age-appropriate MELD/PELD score showed near-chance performance (0.513)—consistent with the limitation of using a listing-allocation tool for postoperative outcome prediction; (2) serial biomarker trajectories exhibited a consistent crossover pattern across multiple parameters that should be regarded as hypothesis-generating; (3) the INR×lactate product—a novel data-driven composite—achieved an apparent AUROC of 0.981 as a standalone metric, derived post hoc from this cohort; (4) the lactate-to-albumin ratio demonstrated a trajectory crossover analogous to INR and ALT, representing, to our knowledge, its first evaluation in this specific setting; and (5) postoperative hypernatremia emerged as an apparent early signal of mortality (AUROC 0.884), linked to the predominance of CNS complications as the leading cause of death. All discriminative estimates are reported with 95% confidence intervals (DeLong for AUROC; Wilson for sensitivity and specificity) in Table 4 and Table 5, and the wide intervals reflect the small number of outcome events (*n* = 11). These findings collectively require external multicenter validation before any clinical translation. It must be emphasized that these findings demonstrate association and predictive discrimination, not causal determination. Several of the strongest postoperative variables identified here—including PRISM-III, CRRT and TPE requirement, hypernatremia, and progressively deranged coagulation—may partly reflect an already evolving catastrophic clinical course rather than independent mechanistic determinants of mortality, and they should be interpreted as concurrent markers of severe early postoperative deterioration rather than as causal drivers.

### 4.1. The Limitations of Pretransplant Severity Scores for Postoperative Mortality Prediction

The strong performance of PRISM-III (AUROC 0.957; cut-off ≥ 14) exceeds its pooled performance in general PICU populations (AUROC 0.84) [8] and represents one of the highest reported discriminative values for this score in pediatric liver transplantation, although confirmation in independent cohorts is needed. Our findings support the observations of Carroll et al. [9] but are discordant with Ferah et al. [10], who found no correlation in 52 recipients from Istanbul. The discrepancy likely reflects our larger sample (140 vs. 52) and our specific focus on 30-day rather than overall mortality. PRISM-III captures the integrated physiological derangement within the first 24 h of PICU admission—precisely the window during which graft function declares itself.

The near-chance performance of the age-appropriate MELD/PELD score (AUROC 0.513; 95% CI 0.333–0.693) is consistent with the hypothesis that pretransplant disease severity scores may be poorly suited for post-transplant mortality prediction in this exploratory analysis. This observation, although intuitively reasonable, requires confirmation in larger external cohorts. After transplantation, short-term survival is likely shaped by graft performance, surgical complications, and the host’s physiological response—domains that PELD was never designed to capture [5,6].

### 4.2. The Biomarker Crossover Pattern

Perhaps the most clinically important observation is the consistent crossover pattern: non-survivors paradoxically exhibited lower pretransplant values of INR, ALT, NLR, and LAR than survivors, yet demonstrated sharp deterioration by 24 h while survivors recovered. This pattern was remarkably consistent across INR, ALT, lactate, and LAR trajectories, which raises the possibility of a unifying mechanism. However, given the small number of events (*n* = 11), the observation that this consistency arose by chance cannot be excluded, and the pattern should be interpreted as hypothesis-generating until reproduced in independent cohorts.

One possible explanation, which we offer as a hypothesis rather than a confirmed mechanism, is that this crossover may reflect a clinically distinct phenotype among non-survivors: patients with relatively preserved pretransplant hepatic function who subsequently experienced catastrophic postoperative events (primary nonfunction, massive CNS complications, acute rejection). It must be emphasized that our data do not directly demonstrate this phenotype and that alternative explanations—including referral patterns, listing thresholds, ascertainment effects related to small event numbers, and unmeasured confounders—cannot be ruled out. Nevertheless, the apparent observation that baseline values appeared reassuring in patients destined for poor outcomes is consistent with the broader interpretation that pretransplant values alone cannot identify at-risk patients; it is the postoperative trajectory that is more informative for outcome.

### 4.3. Novel Composite Biomarkers: INR×Lactate and Lactate-to-Albumin Ratio

The INR×lactate product at 72 h achieved an AUROC of 0.981—approaching the performance of the three-variable multivariate model (0.989)—using only two laboratory values obtainable in seconds from a standard arterial blood gas and coagulation panel. This composite uniquely integrates graft synthetic function (INR reflects hepatocyte protein synthesis) with metabolic clearance capacity (lactate metabolism is critically dependent on functioning hepatocytes) [12,23]. To our knowledge, INR×lactate has not been previously evaluated in this specific setting as a prognostic index in any transplant population. However, several caveats temper this finding. The composite metric and its cut-off thresholds were derived *post hoc* from the development cohort using the Youden index; both the formulation (INR multiplied by lactate) and the optimal threshold are therefore data-driven constructs and carry an inherent risk of optimistic estimation. They cannot be regarded as ready-to-use clinical decision tools at this stage. Their clinical usability—how an elevated INR×lactate value would specifically alter management beyond what an experienced clinician would already infer from elevated INR or lactate individually—remains to be defined in prospective studies. We propose INR×lactate as a candidate exploratory marker for further investigation, not as a validated decision-support index.

The lactate-to-albumin ratio has recently gained attention as a prognostic marker in adult and pediatric critical care, with a nationwide Japanese cohort study confirming its superiority over individual biomarkers for ICU mortality prediction [14]. In pediatric sepsis, an LAR > 0.5 is associated with microcirculatory derangements and increased mortality [13]. Our findings are exploratory in this regard but appear to extend the utility of LAR to the transplant setting, showing a trajectory crossover pattern (pretransplant LAR 0.59 vs. 0.65, *p* = 0.690; 24-h LAR 1.35 vs. 0.59, *p* < 0.001; AUROC 0.909 [95% CI 0.790–1.000]). This crossover parallels the INR and ALT patterns and is consistent with the hypothesis that LAR may capture a composite of perfusion failure and inflammatory-nutritional deterioration developing *after* transplantation in patients with poor short-term outcomes; this remains hypothesis-generating and requires confirmation in independent cohorts.

### 4.4. Impaired Lactate Clearance as a Marker of Graft Dysfunction

Lactate clearance from 0 to 24 h was negative in non-survivors (−25%), indicating that the transplanted liver was not only failing to clear lactate but was contributing to its accumulation. In survivors, clearance was +47%, consistent with effective graft metabolic function. Golse et al. reported that early lactate clearance predicts initial poor graft function in adult liver transplantation with an AUROC of 0.961 [12], and Kim et al. confirmed that 6-h lactate clearance < 25.8% identifies graft dysfunction with 95.5% sensitivity [24]. Our findings are exploratory in this regard but appear consistent with extending this concept to the pediatric population, suggesting that the trajectory—rather than a single time-point value—may carry additional prognostic information; this hypothesis requires confirmation in independent cohorts.

### 4.5. Postoperative Hypernatremia and CNS Mortality

The development of persistent hypernatremia in non-survivors (148 mEq/L at 24 h vs. 139 mEq/L in survivors; AUROC 0.884) is an exploratory observation with potential mechanistic relevance, although it must be interpreted cautiously given the small number of events and the influence of perioperative interventions on serum sodium (see Section 4.11). A recent UNOS analysis of 8011 pediatric liver transplant recipients demonstrated that pretransplant hypernatremia (150–155 mEq/L) independently increases post-transplant mortality 2.49-fold [25]. Our data extend this observation to the postoperative period: pretransplant sodium was comparable between groups, but non-survivors developed de novo hypernatremia within 24 h.

Several mechanisms are biologically plausible—including therapeutic hypertonic saline administration for suspected cerebral edema, impaired renal free water clearance in the setting of multi-organ dysfunction, and aggressive diuresis—but these explanations remain speculative rather than demonstrated, since detailed hypertonic saline dosing, fluid balance, and specific etiologic data for hypernatremia were not systematically collected in this retrospective cohort. The finding is clinically relevant because CNS complications were the leading cause of 30-day death in our cohort (36.4%). Early recognition of postoperative hypernatremia may prompt earlier neuroimaging and neurocritical care consultation.

### 4.6. Combined Extracorporeal Support as a Mortality Marker

The concurrent requirement for CRRT and TPE identified a subgroup with a 52.9% mortality rate, compared with 1.2% among those receiving neither modality. This combination reflects the convergence of hepatorenal dysfunction (necessitating CRRT) and severe coagulopathy or immunological crisis (necessitating TPE)—a phenotype that appears to represent a clinical inflection point in many cases. While extracorporeal support may be life-saving for individual patients, our data suggest that the need for dual modalities may warrant a structured reassessment of graft function within the context of the overall clinical trajectory.

### 4.7. Model Robustness and Internal Validation

Because ΔINR and ΔALT are defined at 72 h, the combined model is best understood as a 72-h landmark risk model rather than as an immediate post-transplant early-warning tool. A critical concern in developing predictive models with 11 events is overfitting. We addressed this rigorously. Bootstrap resampling demonstrated minimal optimism (0.011), indicating that the apparent AUROC of 0.989 was only marginally inflated. LOO-CV—which provides an unbiased estimate by predicting each patient from a model trained without them—yielded an AUROC of 0.957 with 90.9% sensitivity and 96.9% specificity [22]. The relatively small gap between apparent and cross-validated AUROC suggests that the PRISM-III + ΔINR + ΔALT model is unlikely to be entirely driven by sample-specific noise; however, several important caveats apply. First, the events-per-variable ratio of approximately 3.7 is below the conventionally recommended threshold of 10, even with Firth’s penalization, and this is acknowledged as a major limitation that constrains the precision of effect estimates and the generalizability of the model. Second, internal validation, even when performed rigorously, addresses only the sampling variability of the present cohort and cannot quantify between-center heterogeneity, secular trends, or differences in surgical and critical-care practice. Third, formal calibration metrics (Hosmer–Lemeshow goodness-of-fit, Brier score, smoothed calibration plot) could not be reliably computed in our dataset because the small number of events does not allow stable partitioning of the predicted-probability space; we therefore deliberately refrain from reporting potentially misleading calibration statistics. The model should accordingly be regarded as exploratory until calibration and discrimination are confirmed in an independent external cohort. The three model components are universally available in any PICU and require no specialized assays, which would in principle facilitate such external evaluation. It should also be noted that several of the strongest individual predictors—particularly CRRT and TPE requirement, prolonged mechanical ventilation, postoperative hypernatremia, 72-h coagulation derangement, and acute rejection—likely owe part of their very high AUROCs to proximity to the outcome event, whereas PRISM-III at 24 h, ΔINR, and ΔALT lie further upstream and are therefore more genuinely prognostic.

### 4.8. Two Distinct Mortality Patterns

The exploratory comparison of early (≤30-day) and late (>30-day) deaths revealed fundamentally different pathophysiological trajectories. This distinction has an important conceptual implication: the present multivariable model is phenotype-specific, anchored to acute catastrophic deterioration (CNS complications, primary nonfunction, early coagulation derangement, postoperative hypernatremia, and acute rejection), and is not intended for patients whose 30-day mortality follows the late, sepsis-dominated trajectory. Early deaths were driven by acute catastrophic events: CNS complications and primary nonfunction predominated, with acute rejection in 45% of cases and persistently deranged coagulation at 72 h (INR 2.4 vs. 1.6; *p* = 0.001). Late deaths were dominated by prolonged sepsis (78%), with longer mechanical ventilation (27 vs. 14 days; *p* = 0.017) and relatively recovered hepatic function. This descriptive distinction, although based on small numbers, may inform future research directions: prospective studies could test whether early-mortality risk is preferentially modifiable by neuroprotective strategies and aggressive management of graft dysfunction within the first 72 h, whereas late-mortality risk may be more closely linked to sustained infection control and antimicrobial stewardship. These hypotheses are not directly demonstrated by the present data and would require dedicated prospective evaluation. It should also be noted that, although acute rejection was significantly more frequent in non-survivors (45.5% vs. 7.8%) and was associated with substantially higher 30-day mortality (33.3% vs. 4.8%), it was not retained in the multivariable model because the events-per-variable constraint (*n* = 11 events, three variables) precluded inclusion of additional predictors; its absence from the final model therefore reflects a methodological limitation rather than clinical unimportance, and acute rejection should continue to be regarded as an important early postoperative event in this population.

### 4.9. Comparison with Previous Studies

Our study addresses several gaps left by previous work. Cho et al. [3] identified preoperative ICU admission and reoperation as predictors of 30-day mortality in 101 infants but did not evaluate postoperative severity scores or serial biomarkers. Ciria et al. [15] developed a composite score (peak AST × day-2 INR × day-7 bilirubin) for 30-day graft loss prediction (AUROC 0.774) in 422 pediatric recipients—but focused on graft loss rather than patient mortality and did not incorporate ICU severity scores or composite ratios. Our trajectory-based approach, incorporating dynamic composite markers and internal validation, represents a methodological advance over both studies. The INR×lactate product (AUROC 0.981), which requires no scoring system at all, outperforms all previously reported individual predictors in this clinical context.

### 4.10. Clinical Implications

Our findings should be regarded as exploratory and hypothesis-generating, and they are not intended to support immediate changes in clinical decision-making. We do not propose a clinical decision rule. Rather, the present analysis outlines a research roadmap that future prospective multicenter studies could test: namely, whether the combination of an early postoperative severity score (PRISM-III within 24 h), short-interval changes in graft synthetic function (ΔINR), short-interval changes in transaminases (ΔALT), and a simple coagulation–perfusion composite (INR×lactate) can identify, in the early postoperative period, patients at the highest short-term risk after pediatric liver transplantation. The cut-offs reported throughout this manuscript were derived *post hoc* from a single small cohort and are not generalizable thresholds. Any prospective evaluation should pre-specify candidate thresholds (informed by but not identical to those reported here), pre-register analytic plans, and quantify both discrimination and calibration in an independent population before any consideration of clinical adoption.

### 4.11. Limitations

Several limitations merit acknowledgment. First, the single-center retrospective design limits generalizability. Second, despite penalized regression and internal validation, the small number of events (*n* = 11) constrains the complexity of multivariate models. The events-per-variable ratio of approximately 3.7 is below the conventionally recommended threshold of 10, even with Firth’s penalization, and is acknowledged as a major limitation. The absence of external validation is the single most important constraint of this work; the high apparent and internally validated AUROC values reported here should not be interpreted as evidence of generalizable performance, and external validation in an independent multicenter cohort is essential before any clinical application can be considered. Third, the eight-year study period introduces potential temporal confounding from evolving surgical techniques and immunosuppressive protocols. Fourth, the composite biomarkers (INR×lactate, LAR) require prospective evaluation with predefined cut-offs before they can be recommended for clinical decision-making. Fifth, the study did not evaluate donor-specific antibodies, graft steatosis, or detailed immunosuppressive drug levels, which may influence early outcomes.

Sixth, our cohort included 12 adolescent patients (≥12 years, 8.6%) scored by MELD rather than PELD. Although PELD and MELD share some components (INR, bilirubin) but differ in others (PELD incorporates albumin and growth failure; MELD incorporates creatinine), we analyzed them as a single variable for simplicity. This pooling is methodologically defensible because no deaths occurred among the 12 MELD-scored patients, meaning the reported AUROC reflects PELD performance alone. A sensitivity analysis restricted to PELD-scored patients (*n* = 128) yielded comparable results (AUROC 0.524 vs. 0.513), confirming the near-chance performance of pretransplant severity scoring for 30-day post-transplant mortality. Nevertheless, external validation should prospectively evaluate these age groups separately.

Seventh, although several potentially important confounder domains were addressed in our study design—all transplants were performed using living-related donor grafts with confirmed ABO compatibility, all surgeries were carried out by a single dedicated surgical team using a standardized institutional technique, and all recipients received a uniform basiliximab induction plus tacrolimus, mycophenolate mofetil, and tapered prednisolone maintenance protocol that remained unchanged throughout the study period—other confounders were not captured in detail and could still influence both biomarker trajectories and early mortality. These include detailed donor characteristics not retrievable from our records (graft-to-recipient weight ratio, donor steatosis, cold and warm ischemia times), intraoperative variables (blood loss, vascular clamping time, transfusion volume; such perioperative interventions may also transiently influence early biomarker measurements, particularly INR, lactate, sodium, and platelets at the 0-h time point), and the case-by-case clinical thresholds at which CRRT and TPE were initiated. Because the latter decisions were guided by individual clinical judgment rather than fixed numerical criteria, inter-clinician variability cannot be excluded and may have shaped the trajectories of sodium, lactate, and coagulation parameters. The reported associations should not be assumed to hold uniformly across institutions with different donor sources (e.g., predominantly deceased-donor programs), surgical practices, or critical-care thresholds; this further motivates external multicenter validation. Eighth, multiple biomarkers and time points were evaluated in parallel without a single pre-specified primary biomarker, which introduces a multiple-testing bias and contributes to optimism in the ROC analysis. Although our principal findings retained statistical significance well below conventional Bonferroni-adjusted thresholds, the reported AUROC values for individual predictors are likely to be optimistic on average, and confidence intervals (now reported in Table 5) are wide. Ninth, formal calibration metrics (Hosmer–Lemeshow goodness-of-fit, Brier score, smoothed calibration curves) could not be reliably computed given the small number of events; we have therefore restricted formal model assessment to discrimination, and we emphasize that high discrimination does not imply adequate calibration. Together, these considerations underscore that the present results are exploratory and should be interpreted as a foundation for prospective, externally validated, multicenter investigation rather than as evidence supporting current clinical use.

### 4.12. Future Perspectives

Three particularly informative next steps emerge from these findings: (i) mechanistic investigation of postoperative hypernatremia through systematic capture of hypertonic saline dosing, fluid balance, and renal sodium handling; (ii) integration of histopathological and immunological data (biopsy-confirmed rejection, donor-specific antibodies) in larger cohorts to test whether they add prognostic value beyond early physiologic trajectories; and (iii) refinement of the landmark window to evaluate whether earlier timepoints (24- or 48-h) preserve discriminative performance, or whether the 72-h anchor is genuinely necessary.

## 5. Conclusions

In this exploratory single-center cohort, dynamic postoperative factors—rather than pretransplant disease severity—appeared to be more informative for 30-day mortality after pediatric liver transplantation. PRISM-III within 24 h showed substantially better discrimination than the pretransplant age-appropriate MELD/PELD score, a finding more reflective of the limited applicability of listing-era severity tools to postoperative outcomes than of a direct contest between competing instruments, and serial biomarker trajectories exhibited a consistent crossover pattern that should be regarded as hypothesis-generating. The INR×lactate product, a novel two-variable composite requiring no specialized assays, achieved very high apparent discrimination (AUROC 0.981) and is proposed as a candidate exploratory marker for further investigation, not as a validated clinical tool. The combined PRISM-III + ΔINR + ΔALT model demonstrated robust internal validation performance (LOO-CV AUROC 0.957); however, given the small number of events (*n* = 11), the absence of formal calibration assessment, the *post hoc* derivation of all reported cut-offs, and the lack of external validation, the present findings should not be used to guide individual clinical decisions. Prospective multicenter external validation in an independent cohort, with pre-specified candidate thresholds and formal calibration assessment, is essential before clinical implementation; until both external validation and calibration have been demonstrated, the present model and the cut-off thresholds reported here should not be used in clinical decision-making.

## Figures and Tables

**Table 1:** Baseline recipient, donor, and transplant-associated characteristics stratified by 30-day mortality.

Variable	Survivors (*n* = 129)	Deceased (*n* = 11)	*p* Value
Age, months, median (IQR)	22 (11–70)	24 (12–50)	0.464
Sex, female, n (%)	67 (51.9)	6 (54.5)	1.000
Weight, kg, median (IQR)	10.0 (7.8–20.0)	8.5 (7.0–12.0)	0.214
Age-appropriate MELD/PELD score, median (IQR)	19 (16–21)	19 (13.5–20)	0.576
Biliary atresia, n (%)	59 (45.7)	5 (45.5)	1.000
Metabolic disease, n (%)	28 (21.7)	3 (27.3)	0.708
Preoperative INR, median (IQR)	1.7 (1.3–2.2)	1.2 (1.0–1.4)	**0.003**
Preoperative total bilirubin (mg/dL)	14.6 (9.2–19.6)	8.6 (0.9–19.4)	0.175
Preoperative albumin, g/dL	3.2 (2.8–3.7)	3.4 (3.0–3.8)	0.352
Preoperative creatinine (mg/dL)	0.3 (0.2–0.4)	0.2 (0.2–0.3)	0.880
Preoperative lactate (mmol/L)	1.9 (1.3–2.5)	2.0 (1.0–2.5)	0.880
Neutrophil-to-lymphocyte ratio	2.6 (1.4–4.8)	1.2 (0.8–1.7)	**0.001**
Donor age, years	36.0 (29.0–43.0)	34 (28–40)	0.412
Donor type			
Living-related donor, n (%)	129 (100.0)	11 (100.0)	—
ABO-compatible graft, n (%)	129 (100.0)	11 (100.0)	—
Operation duration, min	532 (465–610)	465 (420–520)	**0.016**
Preoperative mechanical ventilation, n (%)	13 (10.1)	1 (9.1)	1.000
Preoperative ICU admission, n (%)	25 (19.4)	2 (18.2)	1.000

Data are presented as median (IQR) or n (%). Bold *p*-values indicate statistical significance (*p* < 0.05). Age-appropriate MELD/PELD: PELD calculated for patients < 12 years (*n* = 128); MELD for 12 adolescent patients ≥ 12 years (8.6%). Both scores were analyzed as a single variable because all 30-day deaths occurred in the PELD-scored subgroup. ICU, intensive care unit.

**Table 2:** Postoperative severity, organ support, and complications stratified by 30-day mortality.

Variable	Survivors (*n* = 129)	Deceased (*n* = 11)	*p* Value
PRISM-III, median (IQR)	8.0 (5.0–11.0)	15.0 (13.0–18.0)	**<0.001**
PELOD-2, median (IQR)	4.0 (2.0–7.0)	7.0 (5.0–10.0)	**0.046**
Mechanical ventilation, days	4 (2–8)	14 (9–21)	**<0.001**
PICU length of stay, days	5 (3–9)	14 (6.5–17)	<0.060
Hospital length of stay, days	20 (13–30)	23 (8.5–28)	0.548
CRRT requirement, n (%)	22 (17.1)	9 (81.8)	**<0.001**
TPE requirement, n (%)	34 (26.4)	10 (90.9)	**<0.001**
ECMO requirement, n (%)	5 (3.9)	1 (9.1)	0.394
Acute rejection, n (%)	10 (7.8)	5 (45.5)	**0.002**
Primary nonfunction, n (%)	8 (6.2)	3 (27.3)	**0.042**
Sepsis, n (%)	61 (47.3)	4 (36.4)	0.0544

Data are presented as median (IQR) or n (%). Bold *p*-values indicate statistical significance (*p* < 0.05). PRISM-III, Pediatric Risk of Mortality III; PELOD-2, Pediatric Logistic Organ Dysfunction 2; PICU, pediatric intensive care unit; CRRT, continuous renal replacement therapy; ECMO, extracorporeal membrane oxygenation; TPE, therapeutic plasma exchange.

**Table 3:** Serial Biomarker Trajectories, Delta Values, and Composite Ratios.

Variable	Deceased ≤ 30 d (*n* = 11)	Survivors (>30 d, *n* = 129)	*p*
***Serial laboratory parameters***			
INR—Pretransplant	1.2 (1.1–1.4)	1.7 (1.4–2.0)	**0.003**
INR—0 h	2.4 (1.9–2.6)	2.0 (1.7–2.4)	**0.160**
INR—24 h	3.0 (2.2–3.3)	1.5 (1.4–1.8)	**<0.001**
INR—72 h	2.4 (2.0–2.9)	1.3 (1.1–1.5)	**<0.001**
ALT (U/L)—Pretransplant	63.0 (24.5–92.0)	143.0 (91.0–203.0)	**<0.001**
ALT (U/L)—0 h	295.0 (197.0–450.5)	647.0 (343.0–938.0)	**0.010**
ALT (U/L)—24 h	441.0 (289.5–913.0)	315.0 (197.0–472.0)	**0.068**
ALT (U/L)—72 h	282.0 (218.0–346.5)	151.0 (88.0–207.0)	**<0.001**
Lactate (mmol/L)—Pretransplant	2.0 (1.0–2.5)	1.9 (1.3–2.5)	**0.880**
Lactate (mmol/L)—0 h	3.1 (2.4–5.5)	3.6 (2.5–4.8)	**0.862**
Lactate (mmol/L)—24 h	4.0 (3.0–4.8)	2.0 (1.6–2.6)	**<0.001**
Lactate (mmol/L)—72 h	2.8 (2.2–3.5)	1.2 (0.8–1.6)	**<0.001**
Albumin (g/dL)—Pretransplant	3.4 (2.5–3.8)	2.9 (2.4–3.4)	**0.131**
Albumin (g/dL)—0 h	3.6 (2.9–3.9)	3.1 (2.8–3.4)	**0.168**
Albumin (g/dL)—24 h	2.8 (2.7–3.8)	3.3 (3.1–3.7)	**0.183**
Albumin (g/dL)—72 h	3.0 (2.7–3.4)	3.4 (3.1–3.7)	**0.033**
Sodium (mEq/L) —Pretransplant	136.0 (134.5–139.0)	137.0 (133.0–142.0)	**0.883**
Sodium (mEq/L)—0 h	146.0 (140.5–148.0)	140.0 (137.0–143.0)	**0.007**
Sodium (mEq/L)—24 h	148.0 (144.5–155.5)	139.0 (137.0–143.0)	**<0.001**
Sodium (mEq/L)—72 h	147.0 (146.0–150.0)	139.0 (137.0–143.0)	**<0.001**
***Delta values (pretransplant → 72 h)***			
ΔALT (%)	320.9 (219.0–911.6)	9.8 (−37.9–86.5)	**<0.001**
ΔINR	1.1 (0.6–1.5)	−0.4 (−0.8–0.0)	**<0.001**
ΔTotal bilirubin	0.0 (−8.8–2.5)	−8.2 (−11.9–−4.8)	**0.007**
ΔAlbumin	−0.1 (−0.8–0.5)	0.5 (0.0–1.0)	**0.007**
***Composite biomarker ratios***			
Lactate clearance 0 → 24 h (%)	−25.0 (−75.0–44.4)	47.1 (15.0–61.4)	**0.014**
Lactate clearance 0 → 72 h (%)	12.9 (−45.0–52.2)	65.9 (53.1–78.6)	**0.002**
LAR—Pretransplant	0.6 (0.3–1.0)	0.7 (0.4–0.9)	**0.690**
LAR—24 h	1.4 (0.8–1.5)	0.6 (0.5–0.8)	**<0.001**
LAR—72 h	0.8 (0.6–1.5)	0.4 (0.2–0.5)	**<0.001**
INR×Lactate—24 h	9.9 (6.8–13.3)	3.1 (2.2–4.2)	**<0.001**
INR×Lactate—72 h	6.6 (5.1–7.4)	1.6 (1.1–2.1)	**<0.001**
Platelet ratio (72 h/Pre)	0.4 (0.3–0.6)	0.8 (0.5–1.1)	**0.002**
Fibrinogen/INR—24 h	41.6 (33.5–81.7)	132.2 (95.4–184.7)	**<0.001**
Fibrinogen/INR—72 h	91.9 (66.1–116.0)	203.7 (135.4–265.3)	**<0.001**

Data are presented as median (IQR). Bold *p*-values indicate statistical significance (*p* < 0.05). Positive delta values indicate worsening; positive lactate clearance indicates effective hepatic clearance. Composite biomarker ratios were evaluated as exploratory indices. ΔALT, percent change in alanine aminotransferase; ΔINR, change in international normalized ratio; LAR, lactate-to-albumin ratio (lactate [mmol/L]/albumin [g/dL]); INR×Lactate, coagulation–perfusion composite index.

**Table 4:** Multivariate Penalized Logistic Regression Model and Internal Validation.

Variable	OR	95% CI	*p*
PRISM-III score (per SD)	4.00	1.82–8.79	**0.001**
ΔINR (pretransplant → 72 h)	3.28	1.56–6.89	**0.002**
ΔALT (%) (pretransplant → 72 h)	3.46	1.64–7.30	**0.001**
Firth’s penalized logistic regression was used to address the low events-per-variable ratio (11 events, 3 variables, EPV = 3.7). Candidate predictors were prespecified based on biological plausibility and prior literature.			
***Internal validation***			
**Metric**	**Value**		**Performance**
Apparent AUROC	0.989 (95% CI: 0.971–1.000)		Sensitivity 100% (95% CI 74.1–100); Specificity 93.8% (95% CI 88.2–96.8)
Bootstrap-corrected AUROC	0.978 (1000 iterations)		Mean optimism: 0.011
LOO-CV AUROC	0.957 (95% CI 0.873–1.000)		Sensitivity 90.9% (95% CI 62.3–98.4); Specificity 96.9% (95% CI 92.3–98.8)
CI, confidence interval; AUROC, area under the receiver operating characteristic curve; LOO-CV, leave-one-out cross-validation. External validation in independent cohorts remains essential before clinical implementation.			

**Table 5:** Discriminative Performance of Individual and Composite Predictors.

Predictor	AUROC (95% CI)	Cut-Off	Sensitivity, % (95% CI)	Specificity, % (95% CI)
***Conventional scores***				
PRISM-III	0.957 (0.873–1.000)	14.0	90.9 (62.3–98.4)	90.7 (84.4–94.6)
PELOD-2	0.681 (0.501–0.861)	7.0	54.5 (28.0–78.7)	76.7 (68.7–83.2)
MELD/PELD	0.513 (0.333–0.693)	21.0	90.9 (62.3–98.4)	21.7 (15.5–29.6)
***Delta values***				
ΔINR	0.908 (0.789–1.000)	0.4	90.9 (62.3–98.4)	94.6 (89.3–97.4)
ΔALT (%)	0.927 (0.819–1.000)	96.2	100.0 (74.1–100.0)	78.3 (70.4–84.5)
ΔBilirubin	0.744 (0.572–0.916)	1.3	63.6 (35.3–84.8)	97.7 (93.4–99.2)
***Composite ratios (exploratory)***				
Lactate clearance 0 → 72 h	0.783 (0.618–0.948)	20.0	63.6 (35.3–84.8)	89.9 (83.5–94.0)
LAR 24 h	0.909 (0.790–1.000)	0.8	100.0 (74.1–100.0)	76.7 (68.7–83.2)
LAR 72 h	0.907 (0.787–1.000)	0.6	81.8 (52.3–94.9)	93.0 (87.2–96.3)
INR×Lactate 24 h	0.965 (0.888–1.000)	4.8	100.0 (74.1–100.0)	86.8 (79.9–91.6)
INR×Lactate 72 h	0.981 (0.924–1.000)	3.3	100.0 (74.1–100.0)	95.3 (90.2–97.8)
Platelet ratio	0.786 (0.622–0.950)	0.7	100.0 (74.1–100.0)	53.5 (44.9–61.9)
Fibrinogen/INR 24 h	0.891 (0.763–1.000)	93.4	90.9 (62.3–98.4)	77.5 (69.6–83.9)
Sodium 72 h	0.884 (0.753–1.000)	146.0	81.8 (52.3–94.9)	94.6 (89.3–97.4)

Cut-off values were determined by maximizing the Youden index (sensitivity + specificity − 1). AUROC, area under the receiver operating characteristic curve; PRISM-III, Pediatric Risk of Mortality III; PELOD-2, Pediatric Logistic Organ Dysfunction 2; MELD/PELD, age-appropriate Model for End-Stage Liver Disease/Pediatric End-Stage Liver Disease; LAR, lactate-to-albumin ratio. Composite ratios were evaluated as exploratory indices and require prospective validation with predefined cut-offs. The 95% confidence intervals are now reported directly in the AUROC, sensitivity, and specificity columns above. AUROC CIs were computed using the DeLong method; sensitivity and specificity CIs were computed using the Wilson score method. The wide intervals across most predictors reflect the small number of outcome events (*n* = 11) and reinforce the exploratory nature of these estimates.

## Data Availability

The data presented in this study are available upon reasonable request from the corresponding author. The data are not publicly available due to privacy and ethical restrictions.

## Supplementary Materials

The following supporting information can be downloaded at https://www.mdpi.com/article/10.3390/jcm15114385/s1, Table S1: Comparison of clinical characteristics between early (≤30-day) and late (>30-day) deaths.

## Abbreviations

The following abbreviations are used in this manuscript:

ALT	Alanine aminotransferase
AST	Aspartate aminotransferase
AUROC	Area under the receiver operating characteristic curve
CI	Confidence interval
CNS	Central nervous system
CRP	C-reactive protein
CRRT	Continuous renal replacement therapy
ECMO	Extracorporeal membrane oxygenation
ICU	Intensive care unit
INR	International normalized ratio
IQR	Interquartile range
LAR	Lactate-to-albumin ratio
LOO-CV	Leave-one-out cross-validation
MELD	Model for End-Stage Liver Disease
NLR	Neutrophil-to-lymphocyte ratio
OR	Odds ratio
PELD	Pediatric End-Stage Liver Disease
PELOD-2	Pediatric Logistic Organ Dysfunction 2
PICU	Pediatric intensive care unit
PRISM-III	Pediatric Risk of Mortality III
ROC	Receiver operating characteristic
STROBE	Strengthening the Reporting of Observational Studies in Epidemiology
TPE	Therapeutic plasma exchange
UNOS	United Network for Organ Sharing
