# Supplementary material for: Early Postoperative Predictors of 30-Day Mortality After Pediatric Liver Transplantation: A Trajectory-Based Analysis
Source: J Clin Med. 2026 Jun 5;15(11):4385. doi: 10.3390/jcm15114385 (PMC13257434; doi:10.3390/jcm15114385)
Supplement: Supplementary file 1 [file jcm-15-04385-s001.zip › jcm-4298781-supplementary.pdf]

Supplementary Table S1. Exploratory Comparison of Early (≤30 Days) Versus Late (>30 Days) Mortality

**Variable**	**Early ≤30 d (n = 11)**	**Late >30 d (n = 9)**	**p**
Demographics and severity			
Age (months)	21.0 (9.0–44.0)	50.0 (22.0–143.0)	**0.183**
Weight (kg)	8.3 (7.0–15.0)	18.0 (11.0–21.0)	**0.138**
PRISM-III	15.0 (14.0–16.5)	15.0 (12.0–16.0)	**0.400**
ICU course			
MV duration (days)	14.0 (8.5–17.0)	27.0 (20.0–35.0)	**0.017**
PICU stay (days)	14.0 (6.5–17.0)	16.0 (4.0–45.0)	**0.382**
Hospital stay (days)	23.0 (8.5–28.0)	45.0 (30.0–45.0)	**0.016**
Laboratory (72 h)			
INR 72 h	2.4 (2.0–2.9)	1.6 (1.5–1.6)	**0.001**
Lactate 72 h	2.8 (2.2–3.5)	3.3 (2.0–4.0)	**0.676**
Sodium 72 h	147.0 (146.0–150.0)	142.0 (141.0–145.0)	**0.039**
Day of death	14.0 (8.5–17.0)	52.0 (40.0–240.0)	**<0.001**
Complications			
CRRT	9/11 (82%)	6/9 (67%)	—
CNS complications	4/11 (36%)	1/9 (11%)	
TPE	10/11 (91%)	8/9 (89%)	—
Sepsis	4/11 (36%)	7/9 (78%)	—
Rejection	5/11 (45%)	1/9 (11%)	—
PNF	3/11 (27%)	3/9 (33%)	—

Exploratory analysis; hypothesis-generating. Data are presented as median (IQR) or n/N (%). Small sample sizes preclude formal statistical inference for categorical variables. CRRT, continuous renal replacement therapy; TPE, therapeutic plasma exchange; PNF, primary nonfunction.
